# Role of Cysteines in the Stability and DNA-Binding Activity of the Hypochlorite-Specific Transcription Factor HypT

**DOI:** 10.1371/journal.pone.0075683

**Published:** 2013-10-07

**Authors:** Adrian Drazic, Amelie Tsoutsoulopoulos, Jirka Peschek, Jasmin Gundlach, Maike Krause, Nina C. Bach, Katharina M. Gebendorfer, Jeannette Winter

**Affiliations:** Center for Integrated Protein Science Munich at the Department Chemie, Technische Universität München, Garching, Germany; Université Paris-Diderot, France

## Abstract

Reactive oxygen species are important components of the immune response. Hypochlorite (HOCl) is produced by neutrophils to kill invading microorganisms. The bactericidal activity of HOCl is due to proteome-wide unfolding and oxidation of proteins at cysteine and methionine residues. *Escherichia coli* cells are protected from HOCl-killing by the previously identified dodecameric transcription factor HypT (YjiE). Here, we aimed to unravel whether HOCl activates HypT directly or via a reaction product of HOCl with a cellular component. Bacterial viability assays and analysis of target gene regulation indicate that HypT is highly specific to activation by HOCl and that no reaction products of HOCl such as monochloramine, hydroxyl radicals, or methionine sulfoxide activate HypT *in vivo*. Surprisingly, purified HypT lost its DNA-binding activity upon incubation with HOCl or reaction products that oxidize HypT to form a disulfide-linked dimer, and regained DNA-binding activity upon reduction. Thus, we postulate that the cysteines in HypT contribute to control the DNA-binding activity of HypT *in vitro*. HypT contains five cysteine residues; a HypT mutant with all cysteines substituted by serine is aggregation-prone and forms tetramers in addition to the typical dodecamers. Using single and multiple cysteine-to-serine mutants, we identified Cys150 to be required for stability and Cys4 being important for oligomerization of HypT to dodecamers. Further, oxidation of Cys4 is responsible for the loss of DNA-binding of HypT upon oxidation *in vitro*. It appears that Cys4 oxidation upon conditions that are insufficient to stimulate the DNA-binding activity of HypT prevents unproductive interactions of HypT with DNA. Thus, Cys4 oxidation may be a check point in the activation process of HypT.

## Introduction

Reactive oxygen species (ROS) are formed as a by-product of aerobic respiration and are also generated by the immune system and mucosal barrier epithelia to fight bacterial infections [1]–[3]. An enhanced and uncontrolled overproduction of ROS by the enzyme myeloperoxidase in activated neutrophils is associated with many inflammatory and neurodegenerative diseases [4]. The bactericidal activity of ROS is due to oxidative damage to macromolecules such as DNA, lipids, and proteins, and their accumulation leads to oxidative stress [5], [6]. The extent of damage depends on the type of ROS (for review see ref. [7]). Superoxide, redox-cycling drugs, and hydrogen peroxide (H_2_O_2_) for example, oxidize iron-sulfur clusters [8], [9], thus inactivating iron-sulfur cluster containing enzymes [10], [11]. H_2_O_2_ also oxidizes cysteines and methionines in proteins, thereby mostly inactivating them [10], [12]–[15]. Cysteine residues can be oxidized to sulfenic, sulfinic and sulfonic acids, whereas the sulfur in methionine can be oxidized to generate methionine sulfoxide (Met-SO) or even methionine sulfone [5]. HOCl and chloramines, which are reaction products of HOCl with amine groups, in sharp contrast, are far more reactive and cause mostly non-selective oxidative damage on a proteome-wide scale. Both show high reactivity towards cysteine and methionine residues [5], as well as the ε-amine group of lysine residues [16]. HOCl causes proteome-wide cysteine and methionine oxidation and protein unfolding [17]–[19]. Hydroxyl radicals (HO•) are the most reactive oxygen species known. They are produced in a Fenton type reaction in the presence of H_2_O_2_ or HOCl and iron [20]. Their high cytotoxicity is derived from a high reactivity towards macromolecules causing extensive DNA damage and mutations as well as extensive damage to proteins [5], [6].

Despite the multifaceted effects of different ROS, they have in common that ROS-specific stress responses are activated. Such stress responses are reversibly activated and protect cells from the hazardous effects of the stress (for review see refs. [7], [21]–[23]). A number of proteins have been identified in the past that utilize cysteine thiols to regulate their own activity or play key roles in maintaining cellular redox homeostasis. Examples are the oxidative stress transcription factors OxyR from *E. coli* and yeast Yap1, which employ H_2_O_2_-induced intramolecular disulfide bond formation to activate their function [24], [25]. The regulators HypR from *Bacillus subtilis* and NemR from *E. coli* undergo cysteine oxidation and confer protection against HOCl via the flavin oxidoreductase HypO [26] and de-repression of *gloA* and *nemA* [27]. A further example is the redox-regulated chaperone Hsp33, whose redox-sensing cysteines form a disulfide bond upon severe oxidative stress (such as HOCl stress or combined heat and H_2_O_2_ stress) and whose linker region becomes concomitantly unfolded to activate the chaperone function and prevent proteome-wide aggregation of cellular proteins [17], [28], [29]. Oxidoreductases, i.e. enzymes involved in oxidative protein folding and reduction, such as thioredoxin, glutaredoxin, Dsb proteins, and protein disulfide isomerase utilize cysteines in a Cys-X-X-Cys motif to form mixed disulfides with substrate proteins, thus altering their redox state [30]–[32]. Lastly, enzymes involved in the degradation of H_2_O_2_ and organic peroxides such as AhpC/AhpF (alkyl hydroperoxide reductase), Tpx (peroxiredoxin), and PprX (thiol-dependent peroxidase) employ cysteines as a redox sensor in regulating H_2_O_2_-mediated cell signaling and homeostasis [33]–[35]. The specific regulation of such redox-sensing proteins relies on the high reactivity of the involved cysteines towards oxidation. While ordinary cysteines are oxidized by H_2_O_2_ with a second order rate constant of 2 M^−1^ s^−1^ at neutral pH [36], redox-sensing thiols are quickly and efficiently oxidized with a rate constant of about 10^7^ M^−1^ s^−1^ (e.g., OxyR [37], peroxiredoxins [38]).

Thus, cysteine thiols can function as central switch in regulating protein activity depending on the cellular condition. Given that the role of cysteines in the stability and function of proteins are such manifold, analyzing and understanding their oxidation state is important to understand their potential role in redox regulation. Here we addressed the question which ROS indeed activates the HOCl-specific transcription factor HypT and whether and how the cysteines contribute to the function of HypT. HypT is a LysR-type transcription factor and forms dodecameric, ring-like particles that dissociate in the presence of DNA to dimers and tetramers [39]. HypT is activated by methionine oxidation of three specific methionines (Met123, Met206, Met230) to Met-SO [40]. It regulates a number of genes, some of which are up-regulated (e.g., *metN*, *metB*) while others are repressed (e.g., *fecD*). HypT selectively confers HOCl resistance [39] indicating that HOCl is the activating ROS. Yet, this does not exclude that HOCl, upon entry into the cell, generates a reaction product with a cellular component that serves as the activating species. To pay attention to this possibility, we defined HOCl reaction products that are likely to occur upon HOCl treatment within the cell or in the LB medium used for viability experiments [39]. From all tested compounds, only HOCl activated HypT *in vivo* to confer resistance to *E. coli* cells and regulate target genes indicating that none of the reaction products is likely to serve as activating species for HypT. Despite selective activation *in vivo*, incubation of purified HypT with HOCl or some other ROS caused a loss of DNA-binding activity that correlated with intermolecular disulfide bond formation. To analyze the potential underlying regulatory mechanism, we generated point mutants in which the cysteines were replaced by serine, either individually or simultaneously. A mutant in which all cysteines were replaced by serine (HypT^5C→S^) retained DNA-binding activity but shows a substantially decreased thermal stability and forms tetramers in addition to the typical large oligomers. We found that while Cys150 is important for stability, Cys4 is important for oligomerization of HypT to dodecamers. The observed loss of DNA-binding activity upon oxidation *in vitro* is caused by oxidation of Cys4. Thus, it appears that oxidation of Cys4 prevents DNA-binding of HypT in the case that the oxidizing conditions are insufficient for HypT activation.

## Materials and Methods

### Generation of Mutants

Single and multiple point mutants of HypT were generated by site-directed mutagenesis and the correct sequence verified by DNA sequencing. HypT and mutants were produced in *E. coli* BL21 (DE3) gold cells for strong overexpression and protein purification purposes [39]. These strains contain pET11a-encoded genes: JC15 (*hypT* [39]), AD49 (*hypT^C4S^*), YLe261 (*hypT^C150S^*), YLe262 (*hypT^C178S^*), and AD51 (*hypT^C242S^*) or pET28a-encoded genes: AD76 (*hypT^C25S^*), YL20 (*hypT* ^5C→S^), YL11 (*hypT^quadrupleC4^*), YL5 (*hypT^quadrupleC25^*), AD63 (*hypT^quadrupleC150^*), YL18 (*hypT^quadrupleC178^*), and YL19 (*hypT^quadrupleC242^*). Resulting proteins carry a C-terminal (pET11a) or N-terminal His-tag (pET28a). For moderate overexpression and viability assays, HypT and mutants were produced from pJW2 [41] in *E. coli* C600 *hypT::*Cm cells (KMG214 [39]). Used strains are as follows: YLe143 (pJW2 [39]), KMG229 (*hypT* [39]), AD56 (*hypT^C4S^*), YLe231 (*hypT^C25S^*), AD60 (*hypT^C150S^*), YLe232 (*hypT^C178S^*), AD57 (*hypT^C242S^*), JW468 (*hypT* ^5C→S^), AD66 (*hypT^quadrupleC1^*), AD65 (*hypT^quadrupleC2^*), AD69 (*hypT^quadrupleC3^*), AD67 (*hypT^quadrupleC4^*), and AD68 (*hypT^quadrupleC5^*).

### Analysis of Cellular Viability

For bacterial viability experiments with various ROS, *E. coli* C600 (wild-type) and C600 *hypT* ^−^ (KMG214, [39]) cells were cultivated in LB medium until an OD600 of 0.4–0.5 was reached. Then, cells were either serially diluted in LB medium (1∶10 dilutions) or washed twice with 100 mM sodium phosphate buffer at pH 7.5 and resuspended in 5-fold the original volume. Diluted cells in LB medium were spotted onto LB agar plates containing 0 to 20 mM hydroxyurea (Sigma-Aldrich). Hydroxyurea generates hydroxyl radicals (HO•) within the cell [42] and was thus used as HO• donor. Phosphate buffer washed cells were distributed into 15 ml tubes (1 ml each) and then either HOCl (Sigma-Aldrich; final concentration 0 to 14 µM), monochloramine (NH_2_Cl, final concentration 0 to 16 µM), Met-SO (Sigma-Aldrich; final concentration 0 to 30 mM), or H_2_O_2_ (Sigma-Aldrich; final concentration 0 to 30 mM) added. After 15 min stress treatment, 1 ml 5-fold concentrated LB medium was added; cells were serially diluted in LB medium (1∶30 dilutions) and spotted onto LB agar plates. The number of viable cells was counted and that of unstressed cells set to 100%. NH_2_Cl was generated by adding 1 mM HOCl under continuously mixing to 10 mM ammonium chloride solution buffered in 50 mM NaPi, pH 7.5. The concentration was determined by UV spectroscopy (extinction coefficient ε_252nm_ = 429 M^−1^cm^−1^) and NH_2_Cl used immediately.

For viability assays with C600 *hypT* ^−^ cells carrying pJW2 (empty pBAD22 plasmid) or expressing wild-type *hypT* or mutants, cells were cultivated in LB medium supplemented with arabinose (0.1% w/v) and ampicillin (200 µg/ml). Cultures were diluted from overnight cultures such that an OD600 of 0.4–0.5 was reached after five doublings. The expression yield was very similar for wild-type *hypT* and all mutants as analyzed by western blot using HypT-specific antibodies (87–114% of wild-type). HOCl stress was performed in LB medium supplemented with 3.5 mM HOCl as described [39]. Samples were serially diluted in LB medium (1∶10 or 1∶30 dilutions) and spotted onto LB agar plates.

### Quantitative Real-time PCR (qRT-PCR)

Phosphate buffer washed cells were left untreated or supplemented with either HOCl (final concentration 3 µM), NH_2_Cl (3 µM), hydroxyurea (100 mM), Met-SO (30 mM), or H_2_O_2_ (1 mM). qRT-PCR and sample preparation of stressed samples was performed as described [39]. Viability of cells during the time course of the experiment was 100%.

### Generation of Single-copy yjiE::lacZ Reporter Fusions and Determination of β-galactosidase Activity

Construction of strains JG159 (*E. coli* C600 *lacZ*::*Km*) and JG160 (C600 *lacZ*::*Km hypT*::*Cm*), each containing the translational *metN::lacZ* reporter fusion, was performed as described previously [39] using a PCR fragment starting at 178 bp upstream and ending 31 bp downstream of the translational start site of *metN* (thus containing the *metN* promoter region) and phage λRS45. Phosphate buffer washed cells were left untreated or supplemented with either HOCl (final concentration 3 µM), NH_2_Cl (3 µM), hydroxyurea (100 mM), Met-SO (30 mM), or H_2_O_2_ (1 mM) for 15 min. β-galactosidase activity was assayed as described previously [39].

### Production and Purification of HypT and Mutants

HypT and mutants were produced and purified via HisTrap chromatography as described previously [40]. Afterwards, proteins were desalted using PD10 columns (GE). Purified proteins were stored in storage buffer (10 mM NaH_2_PO_4_ pH 7.5, 400 mM NaCl, 5% (v/v) glycerol) and reduced (1 mM TCEP (tris(2-carboxyethyl)phosphine) or 1 mM DTT (dithiothreitol), 37°C, 1 h) prior to use as indicated. Experiments were performed in storage buffer unless otherwise indicated.

### Electrophoretic Mobility Shift Assay (EMSA)

EMSA was performed in storage buffer containing 158 bp AlexaFluor488-labeled *hypT* DNA at 20 nM, 1 mg/ml BSA, 1 mM TCEP (in reduced samples), and 6 µM HypT (15 µl total volume). EMSA samples were incubated at 25°C for 20 min before adding 2 µl EMSA loading buffer (40 mM Tris pH 8.4, 4 mM EDTA, 0.2% bromphenol blue, 25% glycerol), separation on 6% TBE gels (Invitrogen) and visualization of DNA-protein complexes using ethidiumbromide or by fluorescence (Typhoon Imager 9200 (GE)).

### Oxidation of HypT *in vitro*

HypT was reduced (10 mM DTT, 37°C, 2 h), DTT removed using PD10 columns, and then oxidized with either HOCl (final concentration 25 µM, 25°C, 30 min), NH_2_Cl (25 µM, 37°C, 1 h), H_2_O_2_ (1 mM, 37°C, 1 h), HO• (200 µM, 37°C, 1 h), or Met-SO (1 mM, 37°C, 1 h). HOCl and NH_2_Cl were quenched with methionine (2 mM final concentration) and H_2_O_2_ was quenched with catalase. Samples were buffer-exchanged prior to further analysis. HO• were generated by incubation of 1 mM Fe^2+^SO_4_ with 1 mM H_2_O_2_ (37°C, 1 h). HO• generation was analyzed and concentration determined using p-nitrosodimethylanilin (pNDA [43]); in the above reaction, HO• was generated quantitatively (1 mM). Additionally, the potency of generated HO• in damaging proteins was analyzed. BSA was incubated with Fe^2+^SO_4_, H_2_O_2_, or HOCl (final concentration 100 µM), or Fe^2+^SO_4_ plus H_2_O_2_ (100 µM each; 25°C, 30 min). HOCl and HO• (i.e., Fe^2+^SO_4_ plus H_2_O_2_) caused complete aggregation of BSA consistent with strong protein damage while Fe^2+^SO_4_ and H_2_O_2_ alone did not alter the solubility of BSA (data not shown).

### Non-reducing SDS Gels and Western Blot

Purified and reduced HypT was treated with different ROS as described above. Samples were split and one half was used directly whereas the other half was treated with 1 mM TCEP (25°C, 1 h) in order to re-reduce the protein. SDS sample buffer (with or without β-mercaptoethanol) was added. KMG229 cells grown in LB medium with arabinose to an OD_600_ of 0.5 were treated with either HOCl (final concentration 3 µM and 8 µM), NH_2_Cl (3 µM, 8 µM), hydroxyurea (5 mM, 100 mM), Met-SO (5 mM, 30 mM), or H_2_O_2_ (5 mM, 30 mM) for 15 min. Cell pellets were resuspended in non-reducing sample buffer. Samples were separated on a neutral pH gradient SDS-gel (Serva) and blotted onto PVDF membrane followed by decoration with HypT-specific antibodies.

### Analysis of Soluble and Insoluble Proteins

Expression cultures were harvested and resuspended in lysis buffer (10 mM Tris pH 7.5, 30 mM NaCl, 100 µg/ml lysozyme, HP protease inhibitor (Serva)). Samples were quickly frozen/thawed four times followed by cell disruption using a bead mill (Retsch; 30 s^−1^, 3×2 min) and centrifugation (13,300 rpm, 4°C, 30 min). Soluble/insoluble proteins were analyzed by western blot. Protein bands were quantified using the ImageJ software. To analyze the *in vitro* solubility, proteins (1.5 µM) were incubated in 10 mM NaH_2_PO_4_ pH 7.5, 5% (v/v) glycerol and NaCl added to a final concentration of 100 to 400 mM (37°C for 30 min). Then, soluble/insoluble proteins were separated by centrifugation (13,300 rpm, 4°C, 30 min) and analyzed on a neutral gel (Serva).

### Circulardichroism (CD) Measurements

Far-UV CD-Spectra were recorded using a Jasco-J-715 spectrometer with 6 µM HypT ± TCEP (1 mM, 1 h). CD-Spectra were recorded from 205 to 260 nm at 37°C (20 nm/min), accumulating 6 spectra. To determine the thermal stability of proteins, samples were incubated from 20°C to 80°C (heating rate 20°C/h) and the CD signal was followed at 222 nm. Curves were fitted using a Boltzmann fit (y = A2+(A1–A2)/(1+e^((x−x^^°^^)/dx)^)).

### Analytical Ultracentrifugation (aUC)

aUC analysis was performed as described [39], [40] using 4 to 10 µM protein, detecting absorbance at 280 nm. Values were corrected for buffer (10 mM NaH_2_PO_4_, 400 mM NaCl, 5% glycerin, pH 7.5). The oligomerization state was analyzed by the c(S) method provided with the SedFit software.

### Mass Spectrometry (MS)

HypT at 0.2 mg/ml was treated with H_2_O_2_ (1 mM final concentration, 37°C, 1 h), remaining free thiols modified with iodo acetamide (iam, 1 mM final concentration, 37°C, 5 min), and samples denatured and trypsin or Glu-C digested as described [40]. As control, TCEP-reduced HypT was treated with iam (1 mM final concentration, 37°C, 5 min) to modify all cysteines. After digestion, the H_2_O_2_-treated samples were split, one half was reduced with DTT (3 mM final concentration, 37°C, 2 h) and the other half left untreated in order to detect potential disulfides between cysteines. Samples were desalted via C18 zip-tip (Millipore) and analyzed by MALDI mass spectrometry (Ultraflex TOF/TOF; Bruker Daltonics) using the reflector mode and linear mode enabling to detect masses up to 10,000. ESI-MS/MS analysis was performed as described [40] and evaluation of data was performed using the program MassMatrix (version 2.4.2; [44]). The settings were as follows: protease: trypsin; decoy database: none; fragmentation: CID; variable modifications: N-terminal acetylation, methionine oxidation, methionine sulfone; precursor ion tolerance: ±10 ppm; product ion tolerance: ±0.8 Da; cross link mode: exploratory. Fragment ions resulting from loss of water or ammonia are not included in the tables shown.

## Results

### HOCl but not Chloramines and Hydroxyl Radicals Activate HypT *in vivo*

HypT is a HOCl-specific transcription factor and confers HOCl resistance [39], [40]. To understand the activation of HypT in HOCl-stressed cells, we started to identify the activating species. There is evidence that HOCl can be directly transported into cells [45]. Nevertheless, HOCl potentially reacts with cellular components to generate a reaction product that may activate HypT. Further, previous experiments have been performed with *E. coli* cells stressed with HOCl in LB medium [39], [40]. Bacterial cells and also LB medium contain a variety of macromolecules that are vulnerable to oxidation by HOCl, most notably amine groups and sulfur containing amino acids [5], [46]. Thus, it seemed reasonable that not HOCl directly but a reaction product of HOCl with LB components causes HypT activation. One potential candidate is monochloramine that is generated quickly at physiological conditions upon reaction of amino groups with HOCl (*k_obs_* for α-amino groups: 5×10^5^ M^−1^ s^−1^ [47]). Further, HOCl reacts with iron to form extremely reactive hydroxyl radicals (HO•) [47], making them a potential activator of HypT. Also the sulfur in methionine is quickly oxidized to form Met-SO (*k_obs_*: 4×10^7^ M^−1^ s^−1^ (for HOCl) and 8×10^9^ M^−1^ s^−1^ (for HO•) [5]). Therefore, we considered testing monochloramine (NH_2_Cl), HO•, Met-SO, and HOCl as HypT-activating ROS.

To test activation of HypT by different ROS, we first performed viability experiments with *E. coli hypT* ^+^ and *hypT* ^−^ cells (C600 and KMG214 [39]) in combination with qRT-PCR to analyze target gene regulation. For stress treatment with HOCl, NH_2_Cl, Met-SO, and H_2_O_2_, cells were washed with phosphate buffer to eliminate all LB compounds and enable determining the effective concentration of ROS in killing cells. For treatment with hydroxyurea (to generate intracellular HO•; [42]), cells were either diluted in LB medium and spotted onto hydroxyurea containing LB agar plates (viability assay) or hydroxyurea was added to liquid LB medium (qRT-PCR analysis) (see ref. [42]). H_2_O_2_ stress was performed as a control because it is known to generate intracellular HO• in a Fenton-type reaction that cause extensive DNA damage and compromise bacterial viability [6].

**Figure 1:**
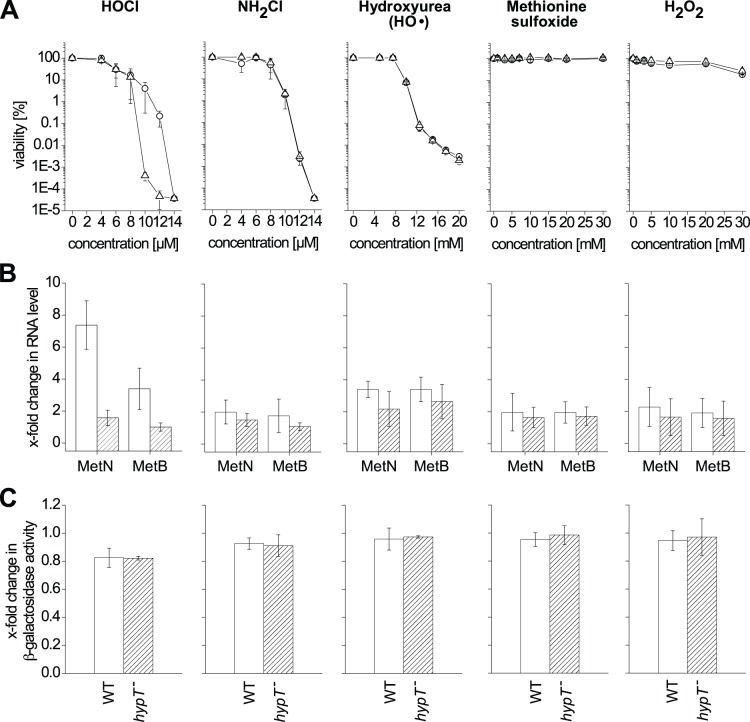
Identification of the activating ROS. A. Viability of C600 (circles) and C600 *hypT* ^−^ (triangles) cells was analyzed in phosphate buffer in the presence of the indicated concentrations of HOCl, NH_2_Cl, hydroxyurea, Met-SO, and H_2_O_2_. Samples were removed after 15 min. Shown is the mean ± standard deviation from three independent experiments. No error bar is visible if the error is very small. B. Analysis of MetN and MetB RNA levels by qRT-PCR. C600 (open bars) and C600 *hypT* ^−^ (striped bars) cells in phosphate buffer were treated with 3 µM HOCl, 3 µM NH_2_Cl, 100 mM hydroxyurea, 30 mM Met-SO, and 1 mM H_2_O_2_ (final concentrations) for 10 min. Cells showed 100% viability during stress. Shown is the mean ± standard deviation from three independent experiments. C. Influence of ROS on MetN translation analyzed by *metN*::*lacZ* reporter fusion. JG159 (C600 *lacZ::*Km; white bars) and JG160 (C600 *lacZ::*Km *hypT::*Cm; striped bars) cells, each containing the translational *metN::lacZ* reporter fusion, in phosphate buffer were treated with 3 µM HOCl, 3 µM NH_2_Cl, 100 mM hydroxyurea, 30 mM Met-SO, and 1 mM H_2_O_2_ (final concentrations) for 15 min. β-galactosidase activity of the *metN::lacZ* reporter fusion was analyzed; shown is the mean ± standard deviation from three independent experiments.

We expect wild-type cells (*hypT* ^+^) to be more resistant to activating compounds than *hypT* ^−^ cells [39]. *hypT* ^+^ and *hypT* ^−^ cells were subjected to stress treatment with HOCl, NH_2_Cl, Met-SO, hydroxyurea, or H_2_O_2_ (Fig. 1A). HOCl was cytotoxic at 10 µM and generated a strong difference in viability between *hypT* ^+^ and *hypT* ^−^ cells, indicating that HOCl activated HypT. Activation of HypT was confirmed by qRT-PCR. MetN and MetB RNA levels were increased upon HOCl stress (maximum at 3 µM HOCl at which cells were 100% viable) in a HypT-dependent manner (Fig. 1B), even though to a lesser extent than observed in LB medium [39]. The cytotoxicity of NH_2_Cl was comparable to HOCl, but no difference in viability of *hypT* ^+^ and *hypT* ^−^ cells and no difference in MetN and MetB RNA levels (up to 8 µM NH_2_Cl) was detected indicating that NH_2_Cl does not activate HypT (Fig. 1A,B). Stress treatment with hydroxyurea was cytotoxic, yet, HO• do apparently not activate HypT because *hypT* ^+^ and *hypT* ^−^ cells showed an indistinguishable viability (Fig. 1A). Further, 100 mM hydroxyurea, at which induction of cell survival responses was demonstrated [42], caused a slight up-regulation of MetN and MetB RNA levels but in a HypT-independent manner (Fig. 1B). Met-SO did neither impair viability of *E. coli* cells nor significantly influence MetN and MetB RNA levels (Fig. 1A,B). Finally, H_2_O_2_ treatment for 15 min did not substantially influence viability and MetN and MetB RNA levels at the applied conditions, demonstrating that generated intracellular HO• are unlikely to cause the observed loss of viability upon HOCl stress. We conclude that HOCl activates HypT in the cell but that NH_2_Cl, HO•, and Met-SO do not activate the transcription factor. Further, we analyzed regulation of *metN* by the various ROS by measuring β-galactosidase activity of a translational *metN*-*lacZ* fusion. *hypT* ^+^ and *hypT* ^−^ cells carrying a chromosomal *metN*-*lacZ* fusion were challenged with ROS as described for qRT-PCR analysis. It should be noted that HOCl is known to inactivate β-galactosidase [48] but H_2_O_2_ and NH_2_Cl do not influence β-galactosidase activity [48], [49]. Accordingly, β-galactosidase activity decreased in our assay by 20% upon HOCl treatment instead of increasing as expected for *metN* from the above qRT-PCR experiments (Fig. 1C; see also [39], [48]). Thus, YjiE-dependent *metN* regulation upon HOCl stress, which showed a maximum at 3 µM in phosphate buffer (see Fig. 1B), could not be analyzed by β-galactosidase assay. However, β-galactosidase activity can be analyzed after treatment with other ROS. β-galactosidase activity did not change significantly upon stress treatment with NH_2_Cl, hydroxyurea, Met-SO, and H_2_O_2_, respectively (Fig. 1C). Since we can exclude a negative effect of H_2_O_2_ and NH_2_Cl on β-galactosidase activity [48], [49], we conclude that MetN translation is not influenced by the tested ROS. This demonstrates that NH_2_Cl, hydroxyurea, Met-SO, and H_2_O_2_ do not activate HypT, which confirms the data obtained by qRT-PCR.

### Oxidation of HypT Leads to Loss of DNA-binding

**Figure 2:**
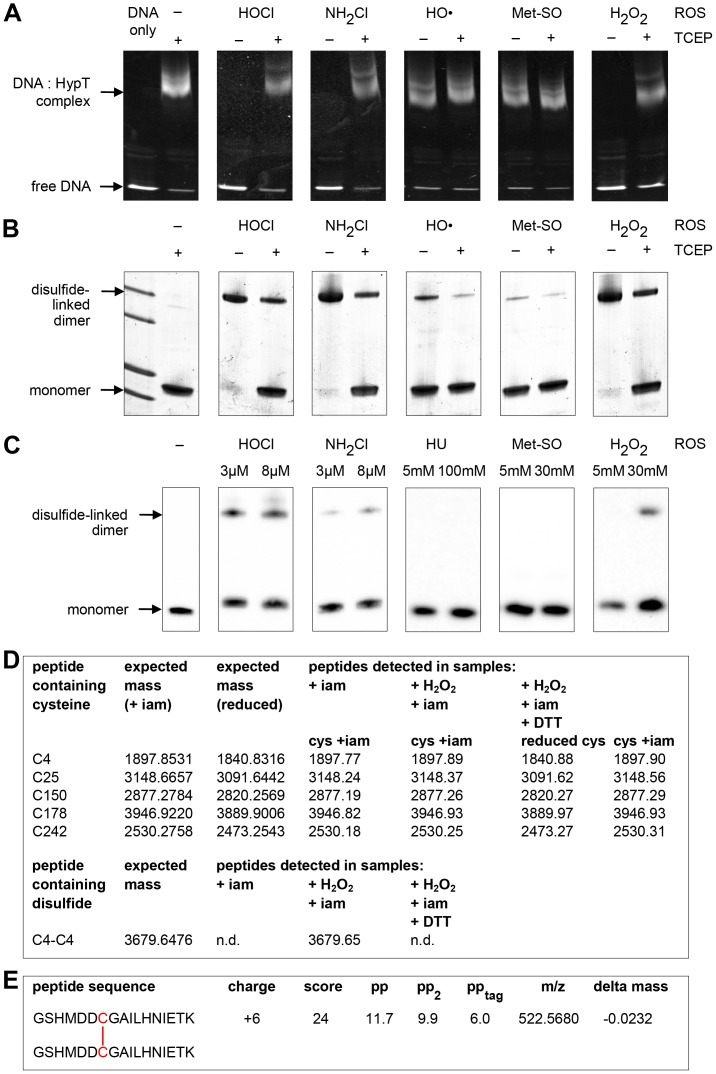
Effect of ROS on HypT DNA-binding activity and cysteines. A. Analysis of the effect of HypT oxidation on DNA-binding activity. Purified HypT was treated with HOCl (25 µM final concentration), NH_2_Cl (25 µM), HO (200 µM), Met-SO (1 mM), and H_2_O_2_ (1 mM, respectively. ROS-treated HypT was incubated with DNA or reduced with TCEP prior to addition of DNA as indicated. Binding of HypT to fluorescent labeled DNA was analyzed by EMSA. Samples were separated on 6% TBE gels and DNA-protein complexes visualized using fluorescence detection. Free DNA and DNA in complex with HypT are indicated. Please note that the loss of DNA-binding is reversed upon reduction. Shown are the results of one representative experiment. B. ROS-treated HypT as in (A) was analyzed by non-reducing SDS-PAGE. Disulfide-linked dimer formation is visible at ca. 64 kDa, which was partially reduced to monomers upon treatment with TCEP. Please note that complete reduction was achieved upon boiling of samples in SDS sample buffer with β-mercaptoethanol. C. *hypT* expressing cells (KMG229) in phosphate buffer were treated with the indicated ROS for 15 min, samples removed and analyzed by non-reducing SDS-PAGE and western blot using HypT-specific antibodies. D. Maldi MS analysis of cysteines and disulfides in H_2_O_2_-treated HypT. H_2_O_2_- and iam-treated HypT was denatured and digested with trypsin or Glu-C. The sample was split and one half was reduced, thus reducing potential disulfides, while the other half was left non-reduced, thus preserving potential disulfides. As control, HypT was reduced and then treated with iam. The five HypT cysteines (reduced or iam-modified) detected in the control, non-reduced, and reduced samples are given in the upper panel. The expected mass of all potential disulfides and the detected disulfides are given in the lower panel. Please note that Cys25 (reduced and iam-modified) and the Cys25-Cys25 disulfide can only be detected in Glu-C digested samples. n.d., not detected. The detected Cys4-Cys4 disulfide is given in the lower panel. E. ESI-MS/MS analysis of HypT after treatment with H_2_O_2_. HypT was H_2_O_2_-treated, denatured, and digested with trypsin. The sample was split and one half was reduced while the other half was left non-reduced, thus preserving potential disulfides. Samples were analyzed by ESI-MS/MS analysis and evaluated using the program MassMatrix. The peptide sequence, charge, scores and mass variation are given; fragment ions are given in Table 1. No other disulfides were detected in HypT and no disulfide was detected in the reduced sample.

Next, we analyzed the effect of HOCl, NH_2_Cl, HO•, Met-SO, and H_2_O_2_ on the DNA-binding activity of purified HypT. HypT was incubated with the respective ROS and then binding to AlexaFluor488-labeled *hypT* promoter DNA [39] was analyzed by EMSA. HypT forms DNA-protein complexes, thus shifting DNA to higher molecular weight. As analyzed by fluorescence anisotropy [39], the size of DNA:HypT complex depends on the activation state of HypT. HypT isolated from HOCl-stressed cells showed a strong and *in vitro* HOCl-oxidized HypT showed a very low fluorescence anisotropy signal compared to HypT purified from unstressed cells [39]. EMSA should enable us to visualize the effect of ROS on the general DNA-binding ability of HypT and to differentiate between HypT that is able to bind to DNA and HypT that does not bind to DNA independently of the activation state. Reduced HypT showed DNA-binding activity that was visible as a shift in molecular weight (Fig. 2A, left panel). Upon ROS treatment the DNA binding activity is expected to be altered; we expect a complete shift of DNA (i.e., no free DNA should remain), a complete loss of DNA binding, or a shift to higher molecular weights, if the DNA:HypT interaction is altered. We observed that upon incubation with HOCl, NH_2_Cl, and H_2_O_2_, HypT completely lost its DNA-binding activity and DNA remained completely detectable as unbound DNA (Fig. 2A). This was reversible upon reduction of ROS-treated HypT with TCEP suggesting that cysteine oxidation occurred upon such treatment (Fig. 2A). In the presence of HO• (generated from FeSO_4_ and H_2_O_2_) and Met-SO, however, HypT maintained nearly the same DNA-binding activity as reduced HypT (Fig. 2A); we observed a very similar shift of DNA to higher molecular weight and a very similar amount of remaining unbound DNA, indicating that HO• and Met-SO do not oxidize HypT’s cysteines.

To test for cysteine oxidation, we analyzed the above HypT samples on non-reducing SDS gels, which should visualize a potential disulfide-linked dimer at ca. 64 kDa. Treatment of HypT with HOCl, NH_2_Cl, and H_2_O_2_ resulted in almost complete formation of a disulfide-linked dimer that was mostly reduced upon TCEP treatment (Fig. 2B). Full reduction of HypT could be achieved by β-mercaptoethanol treatment and boiling the samples (data not shown). HypT treated with HO• and Met-SO, in contrast, did not form an intermolecular disulfide bond but remained detectable at ca. 33 kDa, corresponding to the monomer (Fig. 2B). We performed MS analysis to identify the cysteine residues involved in disulfide bond formation. HypT was treated with H_2_O_2_ (instead of HOCl to avoid unwanted side reactions), then free thiols were modified with iam, and samples were denatured and digested with trypsin or Glu-C. Afterwards, the sample was split and one half was treated with DTT while the other half was left non-reduced, thus preserving potential disulfide bonds. As control, HypT was reduced and then treated with iam. In the control sample as well as the reduced and non-reduced samples, we detected all five HypT cysteines in the iam-modified state, thus revealing that the modification procedure was successful (Fig. 2D). The import difference between the three samples, however, was that we detected a disulfide formed between two peptides containing Cys4 exclusively in the non-reduced sample (Fig. 2D). The corresponding mass for the Cys4-Cys4 disulfide was not detected in the DTT-treated samples but an increase in intensity of the mass corresponding to the peptide containing reduced Cys4. No other disulfides between any of the cysteines were detected. To confirm the presence of a Cys4-Cys4 disulfide, we analyzed HypT after H_2_O_2_ oxidation and trypsin digest by ESI-MS/MS analysis. Using the program MassMatrix [44], we detected the Cys4-Cys4 disulfide (Fig. 2E, fragmentation ions are given in Table 1). No fragmentation scans were obtained for any other potential disulfides. This confirms the presence of the Cys4-Cys4 peptide in the H_2_O_2_-treated, non-reduced sample and suggests that the disulfide-linked dimer observed upon oxidation of HypT is formed between two Cys4 thiols.

**Table 1:** Fragment ions of one Cys4 peptide of the Cys4-Cys4 peptide (according to MassMatrix).

aminoacid #	b^+5^	b^+4^	sequence	y^+5^	y^++^	y^+^	aminoacid #
1			M	626.88			14
2			D	594.28			13
3			D	571.27			12
4		509.70	C	548.27			11
5			G		548.31		10
6		541.72	A				9
7		569.99	I		484.28		8
8	478.81	598.26	L		427.74	854.47	7
9	506.22	632.52	H		371.20	741.39	6
10	529.03	661.03	N			604.33	5
11	551.65	689.31	I			490.29	4
12	577.45	721.57	E			377.20	3
13	597.66		T			248.16	2
			K				1

To test whether disulfide-linked dimer formation also occurs *in vivo*, we treated *hypT* expressing cells with HOCl, NH_2_Cl, HO•, Met-SO, and H_2_O_2_ (similar to Fig. 1B). Similar to treatment of purified HypT, HOCl, NH_2_Cl, and H_2_O_2_ caused disulfide-linked dimer formation in cells (Fig. 2C). Yet, while HypT was completely present as disulfide-linked dimer upon HOCl, NH_2_Cl, and H_2_O_2_ treatment *in vitro*, dimer formation was only partial *in vivo* (Fig. 2C). This may suggest that complete disulfide bond formation occurs only in the absence of cellular reductases such as thioredoxin or glutaredoxin. Given that intermolecular disulfide bond formation occurs upon HOCl, NH_2_Cl, and H_2_O_2_ treatment both *in vitro* and *in vivo* but only HOCl-stressed cells show HypT-dependent increased viability and target gene regulation, it seems that disulfide-linked dimer formation and activation of HypT are two independent processes. HypT oxidation on cysteine residues is responsible for the loss of DNA-binding *in vitro*. Low DNA-binding of HOCl-treated HypT was observed before [39], yet, selective inactivation of HypT by cysteine oxidation seems counterintuitive. HypT contains five cysteines (at positions 4, 25, 150, 178, 242). Except Cys178, which is partially conserved, cysteine residues in HypT are variable (see alignment in [39]). Therefore, a role of cysteines in a redox-regulatory scheme as observed for the invariant cysteines in Hsp33 [50], OxyR [24], or peroxiredoxins [34] seems unlikely. If not redox-regulation in the classical sense, what else is the role of the cysteines in HypT?

### Cysteines in HypT are Required for Activity *in vivo* and Stability

**Figure 3:**
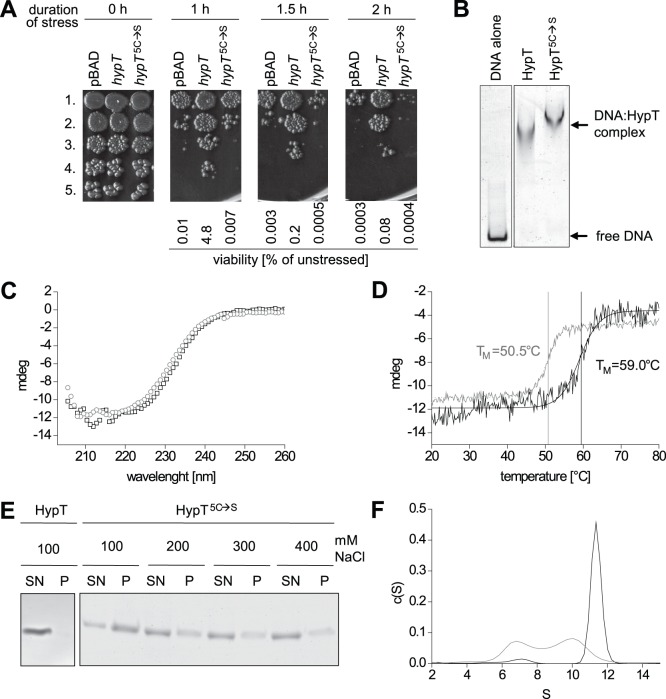
Analysis of the cysteine-free HypT mutant (HypT^5C→S^). A. Viability of C600 *hypT* ^−^ cells carrying an empty plasmid (pBAD) or expressing wild-type *hypT* or *hypT* ^5C→S^ was analyzed at 37°C in the presence of 3.5 M HOCl. Samples were removed at the indicated time points, serially 1∶30 diluted, and spotted onto LB agar plates (1st to 5th dilution). Shown is the result of one representative experiment and the calculated viability relative to unstressed cells. B. Binding of HypT and HypT^5C→S^ to DNA (80∶1 molar ratio) as analyzed by EMSA. DNA-protein complexes were separated on TBE gels and visualized by fluorescence. C. Secondary structure of HypT (black, squares) and HypT^5C→S^ (gray, circles) determined by CD spectroscopy. D. Determination of the thermal stability of HypT (black) compared to HypT^5C→S^ (gray) by observing the thermal transition by CD spectroscopy. E. Salt dependent aggregation of HypT and HypT^5C→S^. Proteins were incubated with the indicated concentrations of NaCl at 37°C, samples centrifuged, and the soluble and insoluble fraction analyzed by SDS-PAGE. SN, soluble fraction; P, pellet fraction. F. Oligomerization state of HypT (black) and HypT^5C→S^ (gray) analyzed by aUC in sedimentation velocity runs. The large particles correspond to a dodecamer while the 6.8 S species represents a tetramer.

In order to directly analyze the role of the cysteines we generated a mutant where all five cysteines were replaced by serine (HypT^5C→S^) and analyzed its ability to confer HOCl resistance. HypT^5C→S^ conferred the same low HOCl resistance as the strain carrying an empty pBAD vector indicating that the cysteines are required for the *in vivo* activity of HypT (Fig. 3A). Next, we purified HypT^5C→S^ and compared its functional and biophysical properties to that of HypT. Both, HypT and HypT^5C→S^ were able to bind to DNA as analyzed by EMSA suggesting that the cysteines are not essential for the DNA-binding activity (Fig. 3B). HypT^5C→S^ and HypT showed a slightly different extent of α-helices according to CD analysis, indicating that the substitution of the cysteines caused detectable changes in secondary structure (Fig. 3C). Further, the thermal stability of HypT^5C→S^ was greatly reduced compared to HypT. According to the thermal transition analysis by CD following the change in signal at 222 nm, HypT^5C→S^ showed an 8.5°C lower midpoint of transition compared to HypT (50.5°C vs. 59.0°C; Fig. 3D). The reduced stability of HypT^5C→S^ became also apparent when its aggregation propensity was determined in dependence of the salt concentration. While the wild-type protein remained soluble even at low salt concentrations (100 mM NaCl; Fig. 3E, left panel), HypT^5C→S^ showed pronounced aggregation that negatively correlated with the salt concentration. HypT^5C→S^ aggregated almost completely during incubation at 100 mM NaCl (Fig. 3E, right panel). Differences between HypT^5C→S^ and HypT were even stronger pronounced when we analyzed their oligomerization state by aUC. We observed that deletion of the cysteines drastically altered the sedimentation behavior of HypT. While the wild-type protein sedimented with 11.4 S, which corresponds to a dodecamer [39], HypT^5C→S^ sedimented with 6.7 S and 10.0 S (Fig. 3F). The two species likely correspond to a tetramer and a decamer or a dodecamer with slightly different shape compared to the wild-type protein. Thus, the cysteines apparently play an important role in the activity *in vivo*, likely by overall stabilizing HypT and its oligomeric structure.

### Cysteine 150 is Required for Stability of HypT *in vivo*

**Figure 4:**
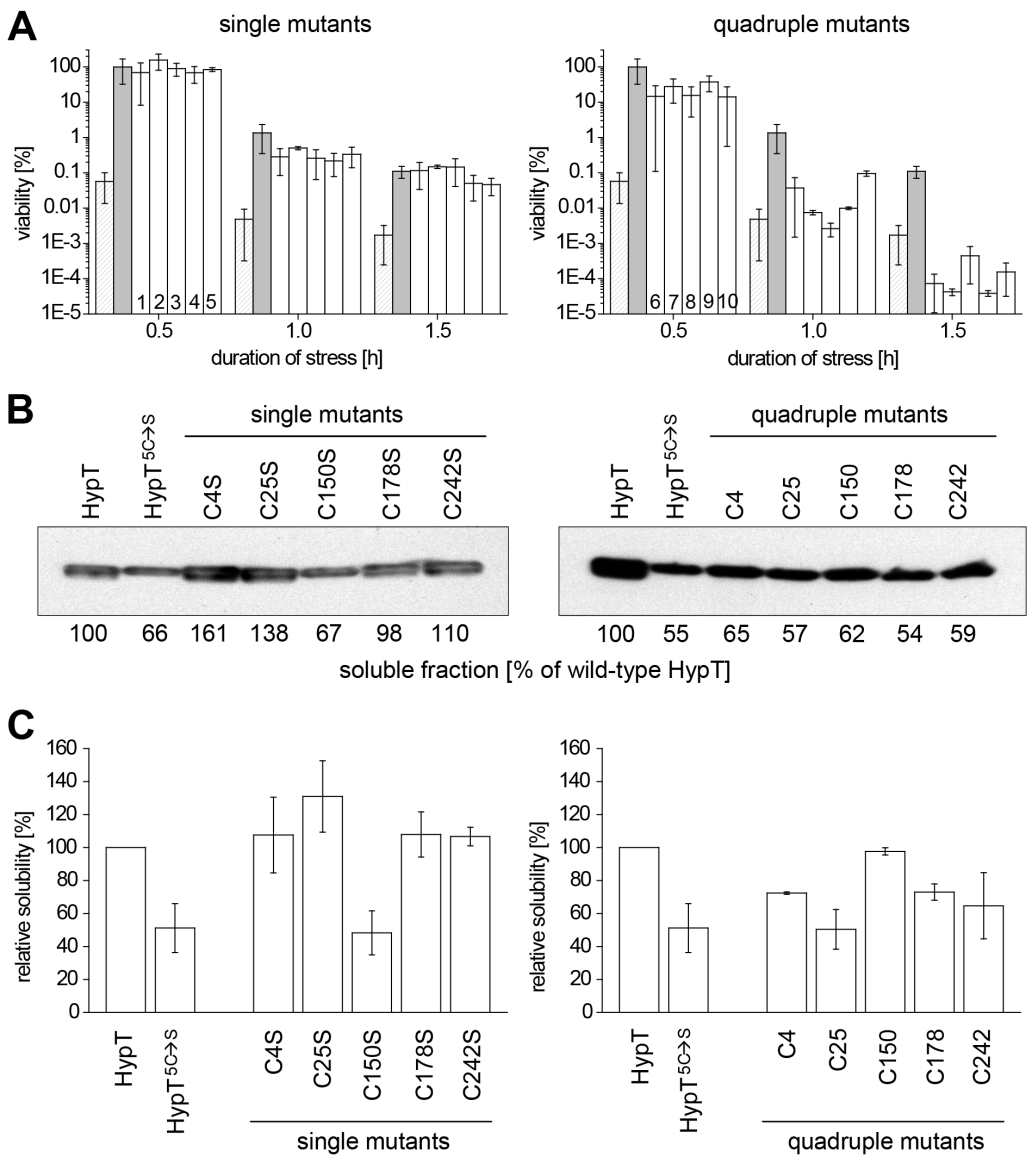
Viability and solubility of single and quadruple cysteine-to-serine mutants of HypT. A. Viability of C600 *hypT* ^−^ cells carrying an empty plasmid (pBAD; striped) or producing wild-type *hypT* (gray), *hypT* single mutants (*hypT^C4S^* (1), *hypT^C25S^* (2), *hypT^C150S^* (3), *hypT^C178S^* (4), *hypT^C242S^* (5)) or *hypT* quadruple mutants (*hypT^quadrupleC4^* (6), *hypT^quadrupleC25^* (7), *hypT^quadrupleC150^* (8), *hypT^quadrupleC178^* (9), *hypT^quadrupleC242^* (10)) analyzed at 37°C in the presence of 3.5 mM HOCl. Samples were removed at the indicated time points and viability analyzed. Shown is the mean ± standard deviation from at least three independent experiments. B. Solubility of HypT and mutants produced in C600 *hypT* ^−^ cells. Cells from (A) were harvested before the addition of HOCl and lysed. Samples were subjected to western blot analysis using HypT-specific antibodies. Band intensities were quantified and are given for soluble protein relative to wild-type HypT. C. Solubility of HypT and mutants overproduced in BL21(DE3) cells. Cells were lysed, samples subjected to western blot analysis using HypT-specific antibodies and band intensities of the soluble fraction quantified. Shown is the mean ± error from at least two independent experiments.

To analyze the individual role of cysteines in HypT, we generated single mutants in which one cysteine was replaced by serine (i.e., HypT^C4S^, HypT^C25S^, HypT^C150S^, HypT^C178S^, HypT^C242S^), and quadruple mutants in which four cysteines were replaced by serine simultaneously thus leaving one cysteine in HypT intact (i.e., quadruple^C4^, quadruple^C25^, quadruple^C150^, quadruple^C178^, quadruple^C242^). First, we analyzed the ability of the mutants to confer HOCl resistance to *hypT* ^−^ cells. The expression level was similar for all mutants (see materials and methods). All single mutants conferred wild-type-like HOCl resistance (Fig. 4A, left panel). The quadruple mutants, in contrast, showed control-like or significantly less HOCl resistance than wild-type HypT and thus seem to be comparably inactive as HypT^5C→S^ (Fig. 4A, right panel). Given the reduced stability of HypT^5C→S^ *in vitro*, we analyzed the amount of soluble HypT or mutant protein in unstressed cells by western blot (Fig. 4B). We quantified the band intensities, set that of wild-type HypT to 100% and related all other values to that. The single mutants HypT^C4S^, HypT^C25S^, HypT^C178S^, and HypT^C242S^ showed a wild-type-like amount of soluble protein (Fig. 4B). However, the soluble protein amount of HypT^5C→S^, the single mutant HypT^C150S^, and all quadruple mutants was much lower than that of wild-type HypT (54–67% of HypT; Fig. 4B). This shows that either substitution of the single Cys150 or simultaneous substitution of more than one cysteine causes reduced solubility of HypT. Based on the observation that the HypT^C150S^ mutant conferred wild-type-like HOCl resistance (see Fig. 4A) but HypT^5C→S^-like solubility, we conclude that the solubility alone does not account for the low HOCl resistance conferred by HypT^5C→S^.

In order to better visualize differences in solubility, we overexpressed *hypT* mutants in BL21(DE3) cells as used for purification purposes and isolated soluble proteins (please note that HypT levels are about five thousand times the HypT level in wild-type cells [39]). We quantified the band intensities from SDS-gels or western blots, set that of HypT to 100% and related all other values to that (Fig. 4C). Similar to the experiments described above, HypT^5C→S^ showed 50% reduced solubility compared to HypT. Further in line, mutants where Cys150 was replaced by serine (i.e., HypT^C150S^, all quadruple mutants except quadruple^C150^) showed reduced solubility (48–73% of HypT). Mutants, however, where Cys150 was still present (i.e., HypT^C4S^, HypT^C25S^, HypT^C178S^, HypT^C242S^, quadruple^C150^) showed wild-type-like or even increased solubility (Fig. 4C). Thus, Cys150 is important for the solubility of HypT in cell lysates.

Given the reduced solubility in cell lysates of some HypT mutants, we next purified the single and quadruple mutants and analyzed their stability and aggregation propensity *in vitro*. The thermal stability of the purified proteins was analyzed by CD and the thermal transition midpoint (T_M_) calculated (Table 2). Mutants where Cys150 was still present (i.e., quadruple^C150^, all single mutants except HypT^C150S^) showed thermal stabilities similar to HypT with T_M_ values above 57°C. Mutants, however, where Cys150 was replaced by serine (i.e., HypT^C150S^, all quadruple mutants except quadruple^C150^) showed thermal stabilities similar to HypT^5C→S^ with T_M_ values below 54°C (Table 2). Thus, Cys150 is not only important for the solubility of HypT in cell lysates but also for the thermal stability of HypT *in vitro*. We conclude that the C150S mutation is most likely the reason for the reduced solubility and stability of the HypT^5C→S^ mutant.

**Table 2:** Thermal stability of HypT and mutants as analyzed by CD.

Mutant	T_M_ [°C]
Wild-type HypT	59.0
HypT^5C→S^	50.5
HypT^C4S^	60.8
HypT^C25S^	58.8
HypT^C150S^	54.3
HypT^C178S^	59.7
HypT^C242S^	57.9
HypT^quadrupleC4^	50.0
HypT^quadrupleC25^	51.0
HypT^quadrupleC150^	57.3
HypT^quadrupleC178^	50.2
HypT^quadrupleC242^	52.5

### Cys4 is Required for HypT Oligomerization to Dodecamers

To further elucidate the role of cysteines in HypT, we next analyzed the oligomerization state of the mutant proteins (Table 3). HypT sedimented with 11.4 S, which corresponds to a dodecamer [39]. The single mutants HypT^C25S^, HypT^C150S^, HypT^C178S^, and HypT^C242S^ sedimented with 10 to 11 S. This corresponds to a decamer or, more likely, a dodecamer with slightly different hydrodynamic radius compared to HypT. Interestingly, HypT^C4S^ formed two species that sedimented with 6.9 S and 10.0 S. This is very similar to HypT^5C→S^ and the quadruple mutants lacking Cys4 (quadruple^C25^, quadruple^C178^, and quadruple^C242^). In contrast, when Cys4 was present as sole cysteine (i.e., quadruple^C4^) the protein sedimented with 11.4 S, just like HypT. Thus, substitution of Cys4 causes the HypT^5C→S^-like oligomerization state of HypT. Of note, quadruple^C150^ sedimented reproducibly as one small species with 7.4 S, this may reflect its decreased stability (Table 3).

**Table 3:** Sedimentation values of HypT and mutants as analyzed by aUC.

Mutant	*s* value	*s* value
	species 1	species 2
HypT	–	11.4
HypT^5C→S^	6.7 (40%)*	10.0 (60%)
HypT^C4S^	6.9 (44%)	10.0 (56%)
HypT^C25S^	–	10.1
HypT^C150S^	–	10.5
HypT^C178S^	–	10.9
HypT^C242S^	–	9.9
HypT^quadrupleC4^	–	11.4
HypT^quadrupleC25^	6.5 (30%)	10.2 (70%)
HypT^quadrupleC150^	7.4	–
HypT^quadrupleC178^	6.2 (25%)	9.1 (75%)
HypT^quadrupleC242^	7.1 (29%)	10.7 (71%)

* Values in brackets represent the ratio of the two species in percent.

### Oxidation of Cys4 Causes Loss of DNA-binding Activity

**Figure 5:**
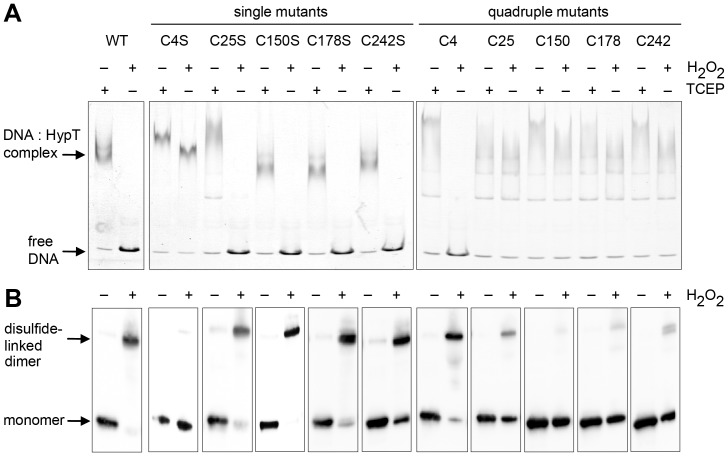
DNA binding and oligomerization of HypT mutants upon oxidation. Analysis of DNA-binding activity (A) and disulfide bond formation (B) of HypT and mutants upon H_2_O_2_ treatment. HypT, single cysteine mutants, and quadruple mutants were either reduced with TCEP (1 mM) or incubated with H_2_O_2_ (1 mM) prior to incubation with DNA. A. Binding of HypT to fluorescent labeled DNA was analyzed by EMSA. Samples were separated on 6% TBE gels and DNA-protein complexes visualized using fluorescence detection. Free DNA and DNA in complex with HypT are indicated. Please note that all proteins are able to bind to DNA in the reduced state. Quadruple mutants show more diffused bands for the HypT-DNA complex, likely because they form less defined oligomers than HypT. Shown are the results of one representative experiment. B. H_2_O_2_-treated samples (without DNA) were analyzed by non-reducing SDS-PAGE. Please note disulfide-linked dimer formation at ca. 64 kDa.

Next we analyzed the DNA-binding activity in the reduced and H_2_O_2_-oxidized state and disulfide-linked dimer formation of the single and quadruple mutants. We chose H_2_O_2_ because it preferentially reacts with cysteine residues in contrast to HOCl that also reacts with other amino acids and may generate unwanted side effects. This should enable us to directly test which cysteine in HypT becomes oxidized *in vitro*. HypT and all mutants were able to bind to DNA and showed formation of DNA-protein complexes under reducing conditions (Fig. 5, TCEP-treated samples). This confirms that cysteines are *per se* not essential for DNA-binding activity of HypT. Upon H_2_O_2_ treatment, however, HypT lost its DNA-binding activity and formed disulfide-linked dimers while HypT^5C→S^ retained DNA-binding activity and was detectable as a single band at ca. 33 kDa (data not shown and Fig. 5A, B). Importantly, mutants lacking Cys4 retained their DNA-binding activity while mutants carrying Cys4 lost DNA-binding activity upon oxidation (Fig. 5A). Coinciding, mutants lacking Cys4 were predominantly detectable as monomer while mutants containing Cys4 formed disulfide-linked dimers upon oxidation (Fig. 5B). The presence of the Cys4-Cys4 disulfide in quadruple^C4^ was confirmed by ESI-MS/MS (data not shown). The absence of disulfides in mutants lacking Cys4 was confirmed by MS analysis, similar to the procedure described for HypT (data not shown). Taken together, the data demonstrates that Cys4 is responsible for intermolecular disulfide bond formation and concomitant loss of DNA-binding activity *in vitro*.

## Discussion

HypT is an oxidative stress transcription factor whose activity depends on the oxidative conditions *in vitro* and *in vivo*. HypT is activated by methionine oxidation to Met-SO [40]. It confers resistance specifically to HOCl-treated *E. coli* cells but not to H_2_O_2_-, and diamide-stressed cells [39], nor to NH_2_Cl-, hydroxyurea-, or Met-SO-stressed cells. It was surprising that neither NH_2_Cl nor HO• activates HypT. Both ROS are highly reactive. They cause, similar to HOCl, complete aggregation of BSA upon treatment *in vitro* (see materials and methods) and decrease bacterial viability and could thus be expected to, at least partially, facilitate HypT activation. We conclude that HypT is a very specific and fine-tuned molecule. *In vitro*, however, several oxidative conditions (HOCl, NH_2_Cl and H_2_O_2_) diminish the DNA-binding activity of HypT. This was unexpected and hampered the identification of the HypT activating ROS *in vitro*. *In vivo*, however, we observed only partial disulfide bond formation, likely because cellular reductases such as thioredoxin, which are up-regulated upon HOCl stress [39], maintain HypT cysteines reduced. So far, we are only able to activate HypT *in vitro* when the cysteines were artificially and reversibly blocked before treatment with HOCl [40]. Since *S*-thiolation of HypT cysteines was not detected [40] this may indicate that cellular reductases are important to keep HypT in a state where specific methionine oxidation occurs and thiol oxidation is prevented. *S*-thiolations such as *S*-glutathionylation [51] and proteome-wide *S*-bacillithiolation [52], [53] are important reversible modifications upon oxidative stress to protect cysteines, especially active site cysteines, from over-oxidation.

We observed a loss of DNA-binding activity upon HOCl, H_2_O_2_, or NH_2_Cl treatment *in vitro* that correlated with intermolecular disulfide bond formation in HypT. HypT contains five cysteines, of which only one is partially conserved. Thus, the cysteines would not be expected to play an important role in HypT function and are not the typical redox sensors as described for OxyR, Hsp33, peroxiredoxins, NemR, OhrR, and HypR [24], [26], [27], [34], [50], [54]. Cysteines in HypT are not essential for DNA-binding *in vitro* as the HypT mutant lacking all cysteines was able to bind to DNA. But cysteines are important for HypT to confer stability and HOCl resistance, even though no individual cysteine was identified to be required for *in vivo* activity. Nevertheless, we determined distinct roles for two of the cysteines in HypT: Cys4 is important for HypT oligomerization to dodecamers and its oxidation leads to loss of DNA-binding while Cys150 is essential for HypT stability. We conclude that the decreased stability and *in vivo* activity of HypT^5C→S^ is derived from the combined substitution of Cys4 and Cys150.

**Figure 6:**
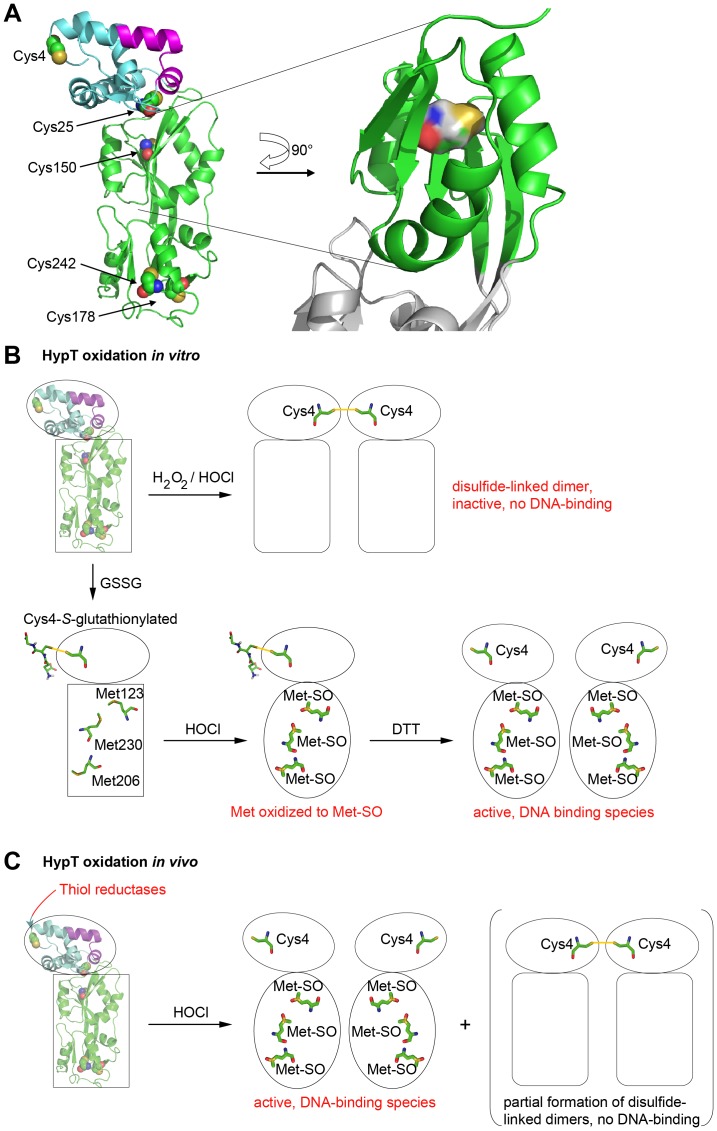
Structural model of HypT and working model. A. Secondary structure prediction of HypT using the prediction software Phyre 2. The cysteines are indicated and the location of Cys150 emphasized. B. Model of HypT function upon oxidative stress *in vitro*. Oxidation of purified HypT leads to disulfide bond formation between Cys4 of one HypT molecule with Cys4 of another HypT molecule and concomitant conformational changes in the N-terminal DNA-binding domain and/or C-terminal domain leading to inhibited DNA binding. Upon reversible protection of cysteine residues by S-glutathionylation, specific activation of HypT by HOCl and generation of the active DNA-binding species occurs. Also here, conformational changes occur in HypT, which potentially positions the DNA-binding domain for efficient DNA-binding. Please note that the exact conformational changes are unknown. C. Model of HypT function upon oxidative stress *in vivo*. HOCl treatment of cells causes methionine oxidation and conformational changes leading to activation of HypT. Cellular thiol reductases maintain HypT cysteines mostly in the reduced state. As a side reaction, partial Cys4-Cys4 disulfide bond formation may occur.

The activity and stability of many proteins relies on cysteines and formation of correct disulfide bonds (e.g., redox-regulated proteins discussed above, proinsulin [55], and lysozyme [56]). Cysteine substitutions are known to destabilize proteins or affect their folding in case the cysteines are engaged in a disulfide bond (e.g., bovine pancreatic trypsin inhibitor, BPTI [57]). *Vice versa*, proteins can be stabilized by introducing a cysteine and enabling disulfide bond formation (e.g., onconase [58]). However, the protein stability may also be altered by cysteine substitution when cysteines are not involved in a disulfide bond. In the case of betaine aldehyde dehydrogenase, substitution of one nonessential cysteine residue by serine resulted in severely reduced stability and higher propensity to dissociation [59]. In rhodanese, mutation of all cysteines that are not required for activity to serine diminished the stability of the enzyme while maintaining its activity [60]. For HypT, it seems likely that replacement of Cys4 and Cys150 by serine leads to altered intra- or intermolecular contacts in HypT, thus, destabilizing the HypT overall structure and oligomer, respectively. This assumption is supported by the modeled structure of HypT, which was generated using the structure prediction software Phyre 2 (Fig. 6A; [61]). There, Cys4 seems to be solvent exposed in a flexible loop in the N-terminal domain and Cys150 seems to be buried in the core of HypT. Substitution of Cys150 by the polar serine may disturb interactions within the core and thus cause loss of stability. *In vitro*, oxidation of Cys4 leads to intermolecular disulfide bond formation and may cause structural changes in the nearby DNA-binding domain, which may block access of the DNA (see working model in Fig. 6B, C). The situation seems to be different *in vivo*, given the presence of thiol reductases, even though also here partial disulfide bond formation occurred. The situation seems also to be different *in vitro* when cysteines were reversibly protected from oxidation by *S*-glutathionylation; this allows for HOCl-mediated methionine oxidation and, upon subsequent reduction of cysteines, high DNA-binding activity [40]. In both such cases, conformational changes may be restricted to the C-terminal domain, allowing for specific methionine oxidation, or conformational changes may occur in the whole HypT molecule and are likely required to bring the DNA-binding domain in the correct position for efficient DNA-binding (Fig. 6B, C). Cys4 may serve as a check point: upon oxidative stress that is insufficient to stimulate HypT DNA-binding (i.e., oxidation *in vitro*), Cys4 becomes oxidized and inhibits DNA-binding to avoid unnecessary regulation of target genes (Fig. 6B, C). This bears some resemblance to the redox-regulated Hsp33, whose chaperone function is activated upon exposure to combined oxidative and unfolding conditions [17], [28]. Either oxidation of cysteine residues or sole unfolding are not sufficient for activation, indicating their simultaneous occurrence as check point to ensure activation upon exactly the stress conditions that require Hsp33 activity [28]. Temporary oxidation and inactivation of HypT shows also some similarities to the peroxiredoxin Tpx1 (thioredoxin peroxidase). Tpx1 degrades peroxides by forming a disulfide bond that is reduced by thioredoxin, yet, Tpx1 is also highly sensitive to peroxide-induced hyperoxidation and inactivation. Hyperoxidation is critical to avoid interaction with thioredoxin, which is thus able to repair oxidized proteins and maintain cell survival [33]. Reversible inactivation of HypT upon certain oxidative stress conditions bears even further resemblance to the temporary inactivation of the chaperone DnaK upon oxidative heat stress [41]. There, DnaK looses its chaperone activity due to loss of cellular ATP, concomitant ATP-depletion of the ATP-dependent DnaK, and heat-induced unfolding of the ATPase domain. This serves to prevent unnecessary and unproductive further ATP hydrolysis by DnaK under non-permissive folding conditions [41].

Taken together, transient oxidation-induced inactivation of HypT may be advantageous to ensure cell survival upon non-specific oxidative stress conditions. This may either be required to ensure lack of HypT-mediated gene regulation or to ensure access of other stress-related factors or transcription factors to specific DNA regions.
